# Supplementary material for: Proteomic Analysis of Prehypertensive and Hypertensive Patients: Exploring the Role of the Actin Cytoskeleton
Source: Int J Mol Sci. 2024 Apr 30;25(9):4896. doi: 10.3390/ijms25094896 (PMC11084483; doi:10.3390/ijms25094896)
Supplement: Supplementary file 1 [file ijms-25-04896-s001.zip › Supplementary Figures S1 and S2.pdf]

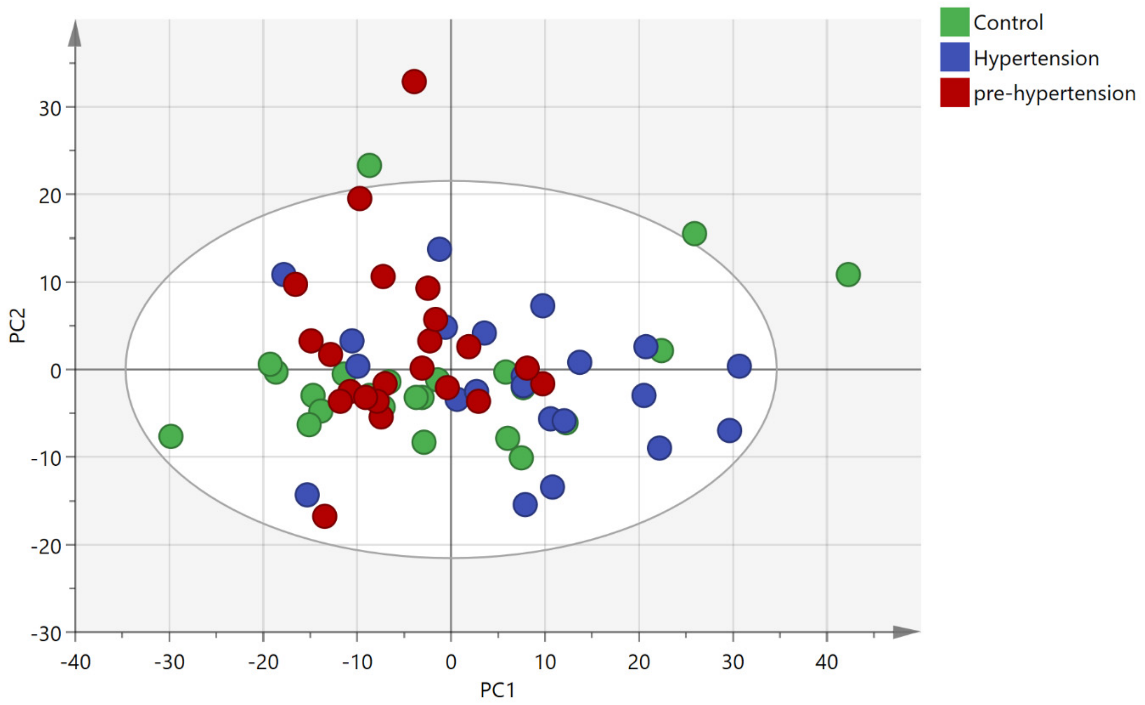


**Figure S1.** Priniciple Component Analysis (PCA) of the particpants in the control, prehypertension and hypertension groups.

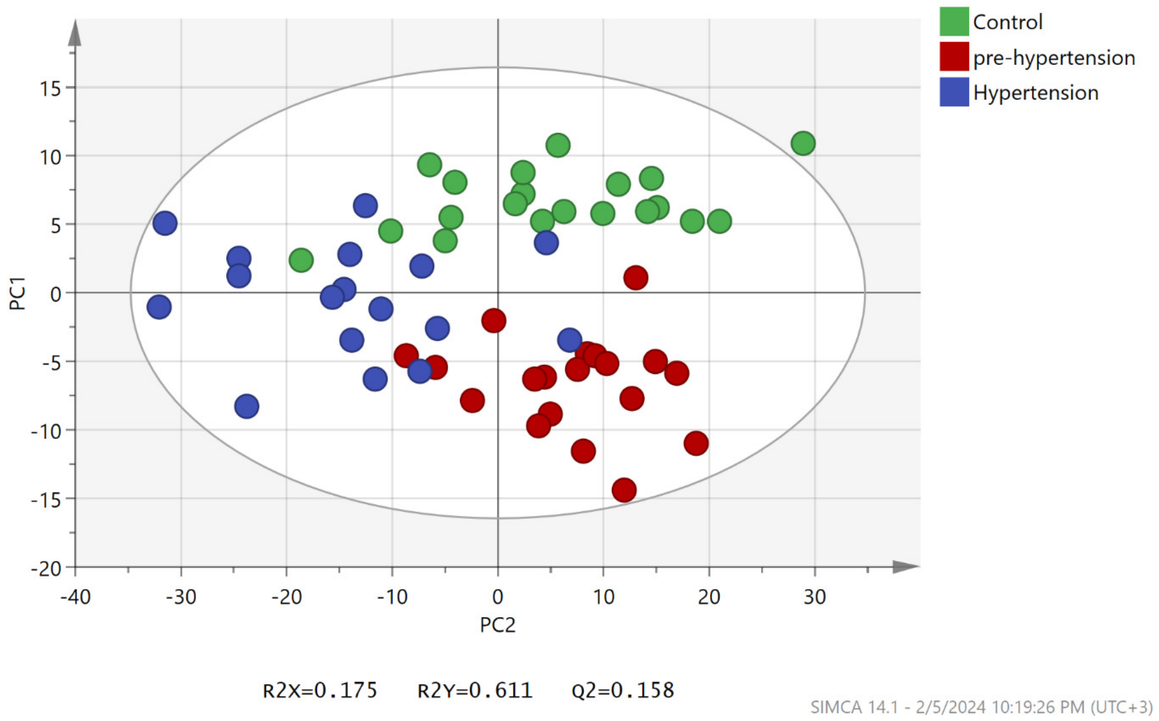


**Figure S2.** Orthogonal partial least squares discriminant analysis (OPLS-DA) scatter plot excluding pateints receiving anti-hypertensive treatment from the study groups.
