# Supplementary material for: Proteomic Analysis of Prehypertensive and Hypertensive Patients: Exploring the Role of the Actin Cytoskeleton
Source: Int J Mol Sci. 2024 Apr 30;25(9):4896. doi: 10.3390/ijms25094896 (PMC11084483; doi:10.3390/ijms25094896)
Supplement: Supplementary file 1 [file ijms-25-04896-s001.zip › Supplementary Table S1.pdf]

Supplementary Table S1. Post hoc analysis conducted after significant ANOVA/Chi square test results for the participants' clinical characteristics.

	Control	Prehypertension	Hypertension	p-value	C vs Pre	C vs Hyp	Pre vs Hyp
**Age**	45.2	51.7	50.2	0.003	<0.001	0.077	0.871
**Hypertension treatment**							
Tablets	0	2	5	0.043	0.23	0.021	0.41
DASH	0	2	5	0.043	0.23	0.021	0.41

C: Control; Pre: Prehypertension; Hyp: Hypertension
