# Supplementary material for: Proteomic Analysis of Prehypertensive and Hypertensive Patients: Exploring the Role of the Actin Cytoskeleton
Source: Int J Mol Sci. 2024 Apr 30;25(9):4896. doi: 10.3390/ijms25094896 (PMC11084483; doi:10.3390/ijms25094896)
Supplement: Supplementary file 1 [file ijms-25-04896-s001.zip › Supplementary Table S2.pdf]

Supplementary Table S2. ANOVA test results showing significantly different proteins between the three groups (*q*-value<0.05). Proteins are represented in terms of their uniprot and gene names.

Uniprot	Gene	*p*-value	*q*-value	Uniprot	Gene	*p*-value	*q*-value
P15531	NME1	<0.001	<0.001	P09326	CD48	0.0018	0.019
P18031	PTPN1	<0.001	<0.001	P42574	CASP3	0.0019	0.019
Q13554	CAMK2B	<0.001	<0.001	P55075	FGF8	0.0020	0.020
Q9UQM7	CAMK2A	<0.001	<0.001	Q9UNP9	PPIE	0.0020	0.020
P49336<br>P24863	CDK8 CCNC	<0.001	0.0013	Q01469	FABP5	0.0020	0.020
Q99714	HSD17B10	<0.001	0.0016	P31946	YWHAB	0.0021	0.020
Q16543	CDC37	<0.001	0.0034	Q9NP79	VTA1	0.0021	0.020
P42226	STAT6	<0.001	0.0049	Q9Y3C8	UFC1	0.0021	0.020
Q04759	PRKCQ	<0.001	0.005	P13693	TPT1	0.0022	0.021
P41743	PRKCI	<0.001	0.0054	Q9UJU6	DBNL	0.0023	0.021
P29353	SHC1	<0.001	0.0055	P60763	RAC3	0.0024	0.021
P15498	VAV1	<0.001	0.0056	P07384<br>P04632	CAPN1<br>CAPNS1	0.0023	0.021
O15530	PDPK1	<0.001	0.0058	Q9UBC2	EPS15L1	0.0025	0.023
P42680	TEC	<0.001	0.006	Q9NYA1	SPHK1	0.0026	0.023
P30419	NMT1	<0.001	0.0096	Q16644	MAPKAPK3	0.0027	0.024
P06744	GPI	<0.001	0.0099	Q08629	SPOCK1	0.0027	0.024
P30101	PDIA3	<0.001	0.0099	O00533	CHL1	0.0028	0.024
Q07817	BCL2L1	<0.001	0.01	Q9UJ70	NAGK	0.0029	0.025
P29350	PTPN6	<0.001	0.010	Q96GD0	PDXP	0.0029	0.025
P31749<br>P31751<br>Q9Y243	AKT1 AKT2<br>AKT3	<0.001	0.011	P05771	PRKCB	0.0030	0.025
P09936	UCHL1	<0.001	0.011	Q8TEU8	WFIKKN2	0.0032	0.026
P16591	FER	<0.001	0.011	Q8N1Q1	CA13	0.0032	0.026
P25098	ADRBK1	<0.001	0.012	P56211	ARPP19	0.0032	0.026
P62993	GRB2	<0.001	0.0125	P17252	PRKCA	0.0033	0.027
P31946	YWHAB	<0.001	0.013	P54646<br>O43741<br>P54619	PRKAA2<br>PRKAB2<br>PRKAG1	0.0036	0.027
Q13557	CAMK2D	<0.001	0.013	P63000	RAC1	0.0036	0.028
P42336<br>P27986	PIK3CA<br>PIK3R1	<0.001	0.013	P62937	PPIA	0.0035	0.028
Q15796	SMAD2	<0.001	0.013	Q14974	KPNB1	0.0035	0.028
Q06187	BTK	<0.001	0.013	P05112	IL4	0.0035	0.028
P07900<br>P08238	HSP90AA1<br>HSP90AB1	<0.001	0.013	Q08431	MFGE8	0.0035	0.028
O00299	CLIC1	<0.001	0.014	P31785	IL2RG	0.0038	0.028
O43323	DHH	<0.001	0.014	P63104	YWHAZ	0.0037	0.028
O95219	SNX4	<0.001	0.014	Q08209<br>P63098	PPP3CA<br>PPP3R1	0.0038	0.028

P10809	HSPD1	<0.001	0.014	Q15465	SHH	0.0039	0.029
Q8N5S9	CAMKK1	<0.001	0.015	P55010	EIF5	0.0042	0.031
P12931	SRC	<0.001	0.016	P84022	SMAD3	0.0043	0.031
Q9P126	CLEC1B	<0.001	0.016	P60900	PSMA6	0.0045	0.032
Q9Y3A5	SBDS	<0.001	0.016	P46108	CRK	0.0045	0.032
P01282	VIP	<0.001	0.016	P54920	NAPA	0.0045	0.032
Q06323	PSME1	<0.001	0.016	Q15056	EIF4H	0.0048	0.034
P41240	CSK	<0.001	0.016	Q8NCW0	KREMEN2	0.0048	0.035
Q7Z3B1	NEGR1	<0.001	0.017	Q16548	BCL2A1	0.0048	0.035
P08238	HSP90AB1	<0.001	0.017	P37802	TAGLN2	0.0052	0.036
Q16539	MAPK14	<0.001	0.017	P50579	METAP2	0.0052	0.037
P36888	FLT3	<0.001	0.017	Q92934	BAD	0.0055	0.037
P22392	NME2	<0.001	0.017	Q01973	ROR1	0.0055	0.038
P67936	TPM4	<0.001	0.017	P26992	CNTFR	0.0054	0.038
P46531	NOTCH1	<0.001	0.017	Q06830	PRDX1	0.0054	0.038
P06241	FYN	<0.001	0.017	P12268	IMPDH2	0.0056	0.038
P05121	SERPINE1	<0.001	0.017	Q9NQU5	PAK6	0.0054	0.038
P07948	LYN	<0.001	0.017	P60174	TPI1	0.0057	0.038
O76074	PDE5A	<0.001	0.017	Q9NP97	DYNLRB1	0.0058	0.039
P31751	AKT2	<0.001	0.017	O95445	APOM	0.0059	0.039
Q9UK53	ING1	<0.001	0.017	P08253	MMP2	0.0060	0.040
P27361	MAPK3	<0.001	0.017	P61960	UFM1	0.0060	0.040
P07948	LYN	<0.001	0.017	P30086	PEBP1	0.0063	0.041
P56199;P05556	ITGA1 ITGB1	<0.001	0.017	P49137	MAPKAPK2	0.0064	0.041
Q9HCR9	PDE11A	<0.001	0.017	P63279	UBE2I	0.0064	0.041
O43488	AKR7A2	<0.001	0.017	P0CG37	CFC1	0.0065	0.041
P48736	PIK3CG	<0.001	0.017	P26038	MSN	0.0066	0.042
P25685	DNAJB1	<0.001	0.017	P17181	IFNAR1	0.0065	0.042
O43320	FGF16	<0.001	0.017	O75556	SCGB2A1	0.0067	0.042
P14618	PKM2	<0.001	0.017	P21217	FUT3	0.0067	0.042
Q9HCN6	GP6	<0.001	0.017	P29401	TKT	0.0075	0.045
Q9NQW7	XPNPEP1	<0.001	0.017	P55854	SUMO3	0.0073	0.046
P07195	LDHB	<0.001	0.017	Q9UMR2	DDX19B	0.0073	0.046
Q9Y5N5	N6AMT1	0.0016	0.017	P63208	SKP1	0.0074	0.046
P40763	STAT3	0.0016	0.018	P30041	PRDX6	0.0077	0.047
P51452	DUSP3	0.0012	0.018	P08581	MET	0.0077	0.047
P40925	MDH1	0.0016	0.018	Q9BY41	HDAC8	0.0078	0.047
P78344	EIF4G2	0.0011	0.018	Q03167	TGFBR3	0.0080	0.048
P30405	PPIF	0.0017	0.018	P20160	AZU1	0.0083	0.049
Q9UNZ2	NSFL1C	0.0011	0.018	Q86YT9	AMICA1	0.0084	0.049
P28482	MAPK1	0.0017	0.018	P00797	REN	0.0085	0.049
P04406	GAPDH	0.0019	0.019	P08684	CYP3A4	0.0084	0.049

P49840 P49841	GSK3A GSK3B	0.0019	0.019	P37840	SNCA	0.0085	0.049
O00170	AIP	0.0019	0.019	O75173	ADAMTS4	0.0086	0.0495
P42224	STAT1	0.0018	0.019				
