# Supplementary material for: Proteomic Analysis of Prehypertensive and Hypertensive Patients: Exploring the Role of the Actin Cytoskeleton
Source: Int J Mol Sci. 2024 Apr 30;25(9):4896. doi: 10.3390/ijms25094896 (PMC11084483; doi:10.3390/ijms25094896)
Supplement: Supplementary file 1 [file ijms-25-04896-s001.zip › Supplementary Table S3.pdf]

Supplementary Table S3: Significantly differentially expressed proteins between controls, pre-hypertension, and hypertension groups. *p*-value <0.05, -0.7< log2 FC >0.7. *FC:* Fold Change.

Hypertenstion | Control

Uniprot	Gene name	Protein name	Log2 FC	*p*-value	*q*-value
**P15498**	VAV1	Proto-oncogene vav	0.94	0.0011	0.07
P18669	PGAM1	Phosphoglycerate mutase 1	0.92	0.0357	0.18
P10809	HSPD1	60 kDa heat shock protein; mitochondrial	0.86	0.0002	0.06
O15530	PDPK1	3-phosphoinositide-dependent protein kinase 1	0.85	0.0006	0.05
Q99714	HSD17B10	3-hydroxyacyl-CoA dehydrogenase type-2	0.84	0.0006	0.05
P16591	FER	Tyrosine-protein kinase Fer	0.83	0.0084	0.11
O95219	SNX4	Sorting nexin-4	0.79	0.0037	0.09
P30405	PPIF	Peptidyl-prolyl cis-trans isomerase F; mitochondrial	0.79	0.0077	0.11
Q06187	BTK	Tyrosine-protein kinase BTK	0.78	0.0102	0.10
Q04759	PRKCQ	Protein kinase C theta type	0.78	0.0026	0.09
P07948	LYNB	Tyrosine-protein kinase Lyn; isoform B	0.76	0.0083	0.11
P06241	FYN	Tyrosine-protein kinase Fyn	0.76	0.0085	0.11
P07948	LYN	Tyrosine-protein kinase Lyn	0.74	0.0070	0.11
P41240	CSK	Tyrosine-protein kinase CSK	0.74	0.0153	0.12
Q15796	SMAD2	Mothers against decapentaplegic homolog 2	0.73	0.0075	0.11
P67936	TPM4	Tropomyosin alpha-4 chain	0.72	0.0099	0.11
P12931	SRC	Proto-oncogene tyrosine-protein kinase Src	0.71	0.0126	0.11
P14618	PKM2	Pyruvate kinase PKM	0.70	0.0145	0.12

Pre-hypertension | Control

Uniprot	Gene name	Protein name	Log2 FC	*p*-value	*q*-value
**P02794 P02792**	FTH1 FTL	Ferritin	1.02	0.03	0.68
P30566	PUR8	Adenylosuccinate lyase	0.74	0.01	0.77

Hypertension | Pre-hypertension

Uniprot	Gene	Protein name	Log2FC	*p*-value	*q*-value
P16591	FER	Tyrosine-protein kinase Fer	1.12	<0.001	<0.001
Q06187	BTK	Tyrosine-protein kinase BTK	1.03	<0.001	<0.001
Q08752	PPID	Peptidyl-prolyl cis-trans isomerase D	1.01	<0.001	0.02
P41240	CSK	Tyrosine-protein kinase CSK	1.00	<0.001	<0.001

P05771	PRKCB	Protein kinase C beta type (splice variant beta-II)	0.99	<0.001	0.01
P15498	VAV1	Proto-oncogene vav	0.98	<0.001	<0.001
Q99714	HSD17B10	3-hydroxyacyl-CoA dehydrogenase type-2	0.96	<0.001	<0.001
P30405	PPIF	Peptidyl-prolyl cis-trans isomerase F; mitochondrial	0.95	<0.001	0.01
P12931	SRC	Proto-oncogene tyrosine-protein kinase Src	0.94	<0.001	<0.001
P18669	PGAM1	Phosphoglycerate mutase 1	0.93	<0.001	0.11
Q04759	PRKCQ	Protein kinase C theta type	0.92	<0.001	<0.001
P14618	PKM2	Pyruvate kinase PKM	0.92	<0.001	0.01
P17252	PRKCA	Protein kinase C alpha type	0.91	<0.001	0.01
O76074	PDE5A	cGMP-specific 3';5'-cyclic phosphodiesterase	0.90	<0.001	<0.001
P67936	TPM4	Tropomyosin alpha-4 chain	0.90	<0.001	<0.001
P07948	LYNB	Tyrosine-protein kinase Lyn; isoform B	0.90	<0.001	0.01
Q15796	SMAD2	Mothers against decapentaplegic homolog 2	0.89	<0.001	0.01
P31946	YWHAB	14-3-3 protein family	0.89	<0.001	<0.001
O15530	PDPK1	3-phosphoinositide-dependent protein kinase 1	0.88	<0.001	0.01
Q9NQU5	PAK6	Serine/threonine-protein kinase PAK 6	0.88	<0.001	0.01
Q9NYA1	SPHK1	Sphingosine kinase 1	0.87	<0.001	0.01
P06241	FYN	Tyrosine-protein kinase Fyn	0.86	<0.001	0.01
P42680	TEC	Tyrosine-protein kinase Tec	0.85	<0.001	<0.001
P07948	LYN	Tyrosine-protein kinase Lyn	0.85	<0.001	0.01
P42574	CASP3	Caspase-3	0.85	<0.001	0.01
O95219	SNX4	Sorting nexin-4	0.84	<0.001	0.01
P62993	GRB2	Growth factor receptor-bound protein 2	0.84	<0.001	<0.001
Q9Y3A5	SBDS	Ribosome maturation protein SBDS	0.84	<0.001	0.01
P78344	EIF4G2	Eukaryotic translation initiation factor 4 gamma 2	0.83	<0.001	0.01
Q13557	CAMK2D	Calcium/calmodulin-dependent protein kinase type II subunit delta	0.83	<0.001	0.01
P22392	NME2	Nucleoside diphosphate kinase B	0.82	<0.001	0.01
O43488	AKR7A2	Aflatoxin B1 aldehyde reductase member 2	0.81	<0.001	0.01
Q15056	EIF4H	Eukaryotic translation initiation factor 4H	0.80	<0.001	0.02
Q8N1Q1	CA13	Carbonic anhydrase 13	0.80	<0.001	0.01
P10809	HSPD1	60 kDa heat shock protein; mitochondrial	0.80	<0.001	0.02
Q13554	CAMK2B	Calcium/calmodulin-dependent protein kinase type II subunit beta	0.79	<0.001	<0.001
O00299	CLIC1	Chloride intracellular channel protein 1	0.78	<0.001	<0.001
P25098	ADRBK1	beta-adrenergic receptor kinase 1	0.78	<0.001	<0.001

P54646<br>O43741<br>P54619	PRKAA2<br>PRKAB2<br>PRKAG1	AMP Kinase (alpha2beta2gamma1)	0.78	<0.001	0.02
Q9NP97	DYNLRB1	Dynein light chain roadblock-type 1	0.77	<0.001	0.01
P49840<br>P49841	GSK3A<br>GSK3B	Glycogen synthase kinase-3 alpha/beta	0.75	<0.001	0.01
P63000	RAC1	Ras-related C3 botulinum toxin substrate 1	0.73	<0.001	0.01
P02775	PPBP	Connective tissue-activating peptide III	0.73	<0.001	0.02
P40763	STAT3	Signal transducer and activator of transcription 3	0.72	<0.001	0.01
P31946	YWHAB	14-3-3 protein beta/alpha	0.71	<0.001	0.01
