# Supplementary material for: Proteomic Analysis of Prehypertensive and Hypertensive Patients: Exploring the Role of the Actin Cytoskeleton
Source: Int J Mol Sci. 2024 Apr 30;25(9):4896. doi: 10.3390/ijms25094896 (PMC11084483; doi:10.3390/ijms25094896)
Supplement: Supplementary file 1 [file ijms-25-04896-s001.zip › Supplementary Table S4.pdf]

Supplementary Table S4: Significant proteins differentially expressed between hypertensive and prehypertensive in the study groups after excluding participants taking hypertension treatment

**Hypertension I Prehypertension**				
**UniProt**	**Gene**	**Protein name**	**Log2FC**	***q*-value**
P16591	FER	Tyrosine-protein kinase Fer	1.29	0.0015
Q06187	BTK	Tyrosine-protein kinase BTK	1.27	0.0017
Q08752	PPID	Peptidyl-prolyl cis-trans isomerase D	1.32	0.006
P41240	CSK	Tyrosine-protein kinase CSK	1.25	0.0016
P05771	PRKCB	Protein kinase C beta type (splice variant beta-II)	1.24	0.0026
P15498	VAV1	Proto-oncogene vav	1.20	<0.001
Q99714	HSD17B10	3-hydroxyacyl-CoA dehydrogenase type-2	1.04	0.0015
P30405	PPIF	Peptidyl-prolyl cis-trans isomerase F; [illegible]	1.15	0.0029
P12931	SRC	Proto-oncogene tyrosine-protein kinase Src	1.14	0.0016
P18669	PGAM1	Phosphoglycerate mutase 1	1.07	0.081
Q04759	PRKCQ	Protein kinase C theta type	1.10	<0.001
P14618	PKM2	Pyruvate kinase PKM	1.15	0.0015
P17252	PRKCA	Protein kinase C alpha type	1.10	0.0037
O76074	PDE5A	cGMP-specific 3';5'-cyclic phosphodiesterase	1.09	0.0012
P67936	TPM4	Tropomyosin alpha-4 chain	1.10	0.0018
P07948	LYNB	Tyrosine-protein kinase Lyn; isoform B	1.02	0.0022
Q15796	SMAD2	Mothers against decapentaplegic homolog 2	1.09	0.0015
P31946	YWHAB	14-3-3 protein family	0.90	0.0013
O15530	PDPK1	3-phosphoinositide-dependent protein kinase 1	1.02	0.0015
Q9NQU5	PAK6	Serine/threonine-protein kinase PAK 6	1.05	0.008
Q9NYA1	SPHK1	Sphingosine kinase 1	1.12	0.002
P06241	FYN	Tyrosine-protein kinase Fyn	1.05	0.0024
P42680	TEC	Tyrosine-protein kinase Tec	0.99	0.0017
P07948	LYN	Tyrosine-protein kinase Lyn	1.08	0.0022
P42574	CASP3	Caspase-3	1.03	0.0026
O95219	SNX4	Sorting nexin-4	1.06	0.0024
P62993	GRB2	Growth factor receptor-bound protein 2	1.005	0.0014
Q9Y3A5	SBDS	Ribosome maturation protein SBDS	1.04	0.0013
P78344	EIF4G2	Eukaryotic translation initiation factor 4 gamma 2	0.99	0.00246
Q13557	CAMK2D	Calcium/calmodulin-dependent protein kinase [illegible]	1.01	0.0014
P22392	NME2	Nucleoside diphosphate kinase B	1.01	0.0013
O43488	AKR7A2	Aflatoxin B1 aldehyde reductase member 2	1.01	0.0016
Q15056	EIF4H	Eukaryotic translation initiation factor 4H	0.98	0.0075
Q8N1Q1	CA13	Carbonic anhydrase 13	0.94	0.0059

P10809	HSPD1	60 kDa heat shock protein; mitochondrial	0.81	0.010
Q13554	CAMK2B	Calcium/calmodulin-dependent protein kinase [illegible]	0.85	<0.001
O00299	CLIC1	Chloride intracellular channel protein 1	0.98	0.0016
P25098	ADRBK1	beta-adrenergic receptor kinase 1	0.96	0.0014
P54646	PRKAA2/B2/G1	AMP Kinase (alpha2beta2gamma1)	0.88	0.012
Q9NP97	DYNLRB1	Dynein light chain roadblock-type 1	0.99	0.0035
P49840	GSK3A/B	Glycogen synthase kinase-3 alpha/beta	0.94	0.0014
P63000	RAC1	Ras-related C3 botulinum toxin substrate 1	0.94	0.0023
P02775	PPBP	Connective tissue-activating peptide III	0.71	0.0942
P40763	STAT3	Signal transducer and activator of transcription 3	0.91	0.0016
P31946	YWHAB	14-3-3 protein beta/alpha	0.90	0.0013
