# Supplementary material for: Proteomic Analysis of Prehypertensive and Hypertensive Patients: Exploring the Role of the Actin Cytoskeleton
Source: Int J Mol Sci. 2024 Apr 30;25(9):4896. doi: 10.3390/ijms25094896 (PMC11084483; doi:10.3390/ijms25094896)
Supplement: Supplementary file 1 [file ijms-25-04896-s001.zip › Supplementary Table S5.pdf]

Supplementary Table S5. Sensitivity and specificity based on ROC analysis of all differentially expressed proteins between the groups.

	Hypertension I Control			Pre-hypertension I Control			Hypertension I Pre-hypertension		
**protein**	**AUC**	**95% CI**	**p-value**	**AUC**	**95% CI**	**p-value**	**AUC**	**95% CI**	**p-value**
**Ferritin**	0.74	0.58 to 0.90	0.010	0.833	0.69 to 0.96	0.00	0.55	0.38 to 0.73	0.527
**PUR8**	0.52	0.32 to 0.72	0.792	0.8	0.64 to 0.95	0.001	0.61	0.43 to 0.80	0.203
**AMPK**	0.76	0.61 to 0.91	0.006	0.628	0.43 to 0.82	0.19	0.70	0.54 to 0.87	0.022
**EIF4H**	0.68	0.52 to 0.84	0.038	0.58	0.40 to 0.75	0.37	0.73	0.58 to 0.88	0.008
**DYNLRB1**	0.65	0.48 to 0.81	0.096	0.62	0.44 to 0.79	0.18	0.75	0.61 to 0.90	0.003
**PPID**	0.66	0.50 to 0.83	0.062	0.59	0.41 to 0.77	0.30	0.75	0.61 to 0.90	0.003
**Cas-3**	0.7	0.54 to 0.85	0.026	0.60	0.43 to 0.78	0.24	0.77	0.63 to 0.91	0.002
**CA13**	0.73	0.58 to 0.89	0.009	0.59	0.41 to 0.77	0.30	0.77	0.63 to 0.91	0.002
**PRKCA**	0.71	0.55 to 0.87	0.016	0.57	0.39 to 0.75	0.41	0.77	0.63 to 0.92	0.001
**PGAM1**	0.74	0.59 to 0.89	0.008	0.53	0.34 to 0.73	0.70	0.78	0.63 to 0.92	0.002
**PAK6**	0.73	0.58 to 0.88	0.010	0.56	0.38 to 0.75	0.45	0.78	0.63 to 0.92	0.001
**SPHK1**	0.71	0.55 to 0.86	0.017	0.58	0.40 to 0.76	0.34	0.78	0.64 to 0.92	0.001
**HSP 60**	0.86	0.75 to 0.98	<0.001	0.51	0.32 to 0.70	0.88	0.78	0.64 to 0.92	0.001
**SNX4**	0.73	0.58 to 0.89	<0.001	0.52	0.34 to 0.71	0.77	0.78	0.64 to 0.93	0.001
**SMAD2**	0.738	0.58 to 0.89	0.0082	0.561	0.38 to 0.74	0.497	0.792	0.64 to 0.93	0.001
**EIF4G2**	0.731	0.57 to 0.88	0.0102	0.573	0.39 to 0.75	0.418	0.796	0.65 to 0.93	0.0009
**AKR7A2**	0.751	0.60 to 0.90	0.0065	0.533	0.34 to 0.72	0.714	0.799	0.65 to 0.93	0.0009
**CAMK2D**	0.720	0.56 to 0.87	0.0146	0.63	0.45 to 0.80	0.159	0.802	0.65 to 0.94	0.0008
**GSK-3A/B**	0.690	0.52 to 0.85	0.0344	0.609	0.42 to 0.79	0.230	0.803	0.66 to 0.94	0.0007
**LYN**	0.727	0.57 to 0.87	0.0118	0.569	0.38 to 0.75	0.449	0.803	0.66 to 0.93	0.0007
**PPIF**	0.75	0.60 to 0.89	0.0056	0.566	0.38 to 0.74	0.465	0.803	0.67 to 0.93	0.0007
**CTAP-III**	0.693	0.53 to 0.85	0.0323	0.612	0.43 to 0.79	0.223	0.804	0.67 to 0.93	0.0007
**LYNB**	0.731	0.58 to 0.88	0.0102	0.576	0.39 to 0.75	0.403	0.805	0.67 to 0.93	0.0006
**GRB2**	0.734	0.58 to 0.88	0.0095	0.6	0.42 to 0.77	0.273	0.811	0.68 to 0.94	0.0005
**NME2**	0.752	0.60 to 0.90	0.0052	0.588	0.40 to 0.76	0.334	0.813	0.67 to 0.94	0.0004
**RAC1**	0.684	0.52 to 0.84	0.0414	0.609	0.42 to 0.79	0.230	0.816	0.68 to 0.94	0.0004
**STAT3**	0.65	0.47 to 0.82	0.1046	0.591	0.40 to 0.77	0.323	0.817	0.67 to 0.95	0.0006
**CSK**	0.715	0.55 to 0.87	0.0167	0.621	0.44 to 0.80	0.183	0.818	0.68 to 0.94	0.0004
**ADRBK1**	0.75	0.60 to 0.89	0.0056	0.578	0.39 to 0.76	0.389	0.818	0.68 to 0.95	0.0004
**VAV**	0.786	0.64 to 0.92	0.0015	0.521	0.33 to 0.70	0.814	0.820	0.68 to 0.95	0.0003
**14-3-3 A/B**	0.706	0.54 to 0.86	0.0219	0.59	0.40 to 0.77	0.330	0.820	0.68 to 0.95	0.0004
**TPM4**	0.715	0.56 to 0.87	0.0167	0.547	0.35 to 0.73	0.601	0.824	0.69 to 0.95	0.0003
**FER**	0.756	0.60 to 0.90	0.0052	0.618	0.43 to 0.79	0.206	0.825	0.68 to 0.96	0.0003
**SRCN1**	0.729	0.57 to 0.88	0.011	0.604	0.42 to 0.78	0.251	0.833	0.70 to 0.96	0.0002
**PKM2**	0.713	0.55 to 0.86	0.0179	0.62	0.43 to 0.80	0.194	0.834	0.70 to 0.96	0.0002
**TEC**	0.743	0.59 to 0.89	0.007	0.6	0.42 to 0.77	0.273	0.835	0.70 to 0.96	0.0002
**PDE5A**	0.711	0.55 to 0.86	0.0192	0.607	0.42 to 0.78	0.240	0.837	0.71 to 0.96	0.0002

**14-3-3 protein family**	0.746	0.59 to 0.89	0.0071	0.578	0.39 to 0.76	0.393	0.837	0.71 to 0.96	0.0002
**CLIC1**	0.690	0.52 to 0.85	0.0344	0.614	0.43 to 0.79	0.210	0.839	0.71 to 0.96	0.0001
**KPCT**	0.769	0.62 to 0.91	0.0032	0.602	0.41 to 0.79	0.267	0.845	0.71 to 0.97	0.0002
**PDPk1**	0.779	0.64 to 0.91	0.002	0.578	0.39 to 0.76	0.399	0.846	0.72 to 0.96	0.0002
**BTK**	0.731	0.57 to 0.88	0.0102	0.63	0.44 to 0.81	0.159	0.847	0.72 to 0.97	0.0001
**CAMK2B**	0.787	0.63 to 0.93	0.0017	0.663	0.49 to 0.83	0.081	0.854	0.71 to 0.98	<0.0001
**SBDS**	0.747	0.59 to 0.89	0.0067	0.588	0.40 to 0.76	0.334	0.859	0.74 to 0.97	<0.0001
**ERAB**	0.778	0.63 to 0.91	0.0023	0.602	0.42 to 0.78	0.267	0.866	0.76 to 0.97	<0.0001
