# Supplementary material for: Proteomic Analysis of Prehypertensive and Hypertensive Patients: Exploring the Role of the Actin Cytoskeleton
Source: Int J Mol Sci. 2024 Apr 30;25(9):4896. doi: 10.3390/ijms25094896 (PMC11084483; doi:10.3390/ijms25094896)
Supplement: Supplementary file 1 [file ijms-25-04896-s001.zip › Supplementary Table S6.pdf]

Supplementary Table S6. DAVID Functional enrichment analysis**.** The gene sets in each of the identified protein clusters were checked for enrichment of KEGG pathway in order to identify biological processes related to the proteins in each cluster.

**Annotation Cluster 1**	**Enrichment Score: 12.27**	**Count**	**p-value**	**Benjamini**
	PI3K-Akt signaling pathway	42	2.80E-17	7.50E-15
	MAPK signaling pathway	36	4.10E-15	4.60E-13
	Pathways in cancer	48	5.10E-15	4.60E-13
	Rap1 signaling pathway	30	2.40E-14	1.60E-12
	Ras signaling pathway	30	5.30E-13	2.80E-11
	Melanoma	16	1.60E-10	3.50E-09
	Focal adhesion	24	6.80E-10	9.60E-09
	Regulation of actin cytoskeleton	24	8.80E-09	1.10E-07
**Annotation Cluster 2**	**Enrichment Score: 7.58**	**Count**	**p-value**	**Benjamini**
	Ras signaling pathway	30	5.30E-13	2.80E-11
	Gastric cancer	18	1.40E-07	1.10E-06
	Hepatocellular carcinoma	14	2.60E-04	8.90E-04
**Annotation Cluster 3**	**Enrichment Score: 5.31**	**Count**	**p-value**	**Benjamini**
	Th17 cell differentiation	16	5.50E-08	5.20E-07
	PD-L1 expression and PD-1 checkpoint pathway in cancer	14	2.40E-07	1.90E-06
	Th1 and Th2 cell differentiation	12	1.50E-05	7.40E-05
	Leishmaniasis	8	2.80E-03	7.10E-03
**Annotation Cluster 4**	**Enrichment Score: 5**	**Count**	**p-value**	**Benjamini**
	Proteoglycans in cancer	27	4.50E-12	2.00E-10
	Fc epsilon RI signaling pathway	17	5.20E-12	2.00E-10
	VEGF signaling pathway	16	7.30E-12	2.40E-10
	HIF-1 signaling pathway	20	1.50E-11	4.30E-10
	EGFR tyrosine kinase inhibitor resistance	17	6.00E-11	1.60E-09
	ErbB signaling pathway	17	1.90E-10	3.90E-09
	Chemokine signaling pathway	24	2.70E-10	5.20E-09
	Chemical carcinogenesis - receptor activation	25	3.50E-10	6.20E-09
	Hepatitis B	22	3.90E-10	6.50E-09
	Focal adhesion	24	6.80E-10	9.60E-09
	Glioma	15	3.10E-09	4.10E-08
	Natural killer cell mediated cytotoxicity	18	1.10E-08	1.30E-07
	Prostate cancer	16	1.20E-08	1.40E-07
	AGE-RAGE signaling pathway in diabetic complications	16	1.90E-08	2.10E-07
	Neurotrophin signaling pathway	17	3.10E-08	3.30E-07
	T cell receptor signaling pathway	16	3.30E-08	3.30E-07
	Growth hormone synthesis, secretion and action	17	3.50E-08	3.50E-07

	B cell receptor signaling pathway	14	8.80E-08	8.10E-07
	Endocrine resistance	15	1.10E-07	9.60E-07
	PD-L1 expression and PD-1 checkpoint pathway in cancer	14	2.40E-07	1.90E-06
	Human immunodeficiency virus 1 infection	21	2.40E-07	1.90E-06
	Kaposi sarcoma-associated herpesvirus infection	20	2.80E-07	2.10E-06
	Pancreatic cancer	13	2.90E-07	2.20E-06
	Toxoplasmosis	15	5.90E-07	4.20E-06
	Fc gamma R-mediated phagocytosis	14	6.60E-07	4.70E-06
	Choline metabolism in cancer	14	7.50E-07	5.10E-06
	Renal cell carcinoma	12	8.40E-07	5.60E-06
	Prolactin signaling pathway	12	9.80E-07	6.30E-06
	Colorectal cancer	13	1.20E-06	7.40E-06
	Non-small cell lung cancer	12	1.30E-06	8.10E-06
	Osteoclast differentiation	15	3.00E-06	1.80E-05
	Breast cancer	16	3.20E-06	1.80E-05
	Apoptosis	15	6.10E-06	3.50E-05
	Sphingolipid signaling pathway	14	6.90E-06	3.80E-05
	Thyroid hormone signaling pathway	14	8.30E-06	4.40E-05
	Human cytomegalovirus infection	19	1.10E-05	5.40E-05
	Platelet activation	14	1.10E-05	5.50E-05
	Chronic myeloid leukemia	11	1.60E-05	7.70E-05
	Cholinergic synapse	13	2.10E-05	1.00E-04
	Hepatitis C	15	3.20E-05	1.40E-04
	Insulin signaling pathway	14	3.20E-05	1.40E-04
	Acute myeloid leukemia	10	3.60E-05	1.50E-04
	Viral carcinogenesis	17	4.30E-05	1.80E-04
	C-type lectin receptor signaling pathway	12	4.80E-05	2.00E-04
	Central carbon metabolism in cancer	10	5.10E-05	2.10E-04
	Platinum drug resistance	10	7.20E-05	2.90E-04
	Phospholipase D signaling pathway	14	7.20E-05	2.90E-04
	Relaxin signaling pathway	13	7.90E-05	3.10E-04
	Cellular senescence	14	1.20E-04	4.70E-04
	Yersinia infection	13	1.40E-04	5.30E-04
	Diabetic cardiomyopathy	16	1.50E-04	5.40E-04
	Estrogen signaling pathway	13	1.50E-04	5.40E-04
	Measles	13	1.60E-04	5.60E-04
	Apelin signaling pathway	13	1.60E-04	5.60E-04
	Hepatocellular carcinoma	14	2.60E-04	8.90E-04
	Gap junction	10	3.10E-04	1.00E-03
	Adrenergic signaling in cardiomyocytes	13	3.30E-04	1.10E-03
	Shigellosis	17	4.00E-04	1.30E-03

	Epstein-Barr virus infection	15	4.70E-04	1.50E-03
	Endometrial cancer	8	5.30E-04	1.70E-03
	MicroRNAs in cancer	19	6.50E-04	2.00E-03
	Progesterone-mediated oocyte maturation	10	9.10E-04	2.80E-03
	Toll-like receptor signaling pathway	10	1.00E-03	3.10E-03
	Influenza A	13	1.10E-03	3.10E-03
	cAMP signaling pathway	15	1.10E-03	3.30E-03
	Chemical carcinogenesis - reactive oxygen species	15	1.20E-03	3.50E-03
	FoxO signaling pathway	11	1.50E-03	4.00E-03
	TNF signaling pathway	10	1.80E-03	4.70E-03
	Aldosterone-regulated sodium reabsorption	6	2.00E-03	5.30E-03
	Autophagy - animal	11	2.50E-03	6.50E-03
	Neutrophil extracellular trap formation	13	2.60E-03	6.60E-03
	Salmonella infection	15	3.50E-03	8.50E-03
	Chagas disease	9	3.70E-03	8.90E-03
	GnRH secretion	7	4.80E-03	1.20E-02
	mTOR signaling pathway	11	5.30E-03	1.20E-02
	Human papillomavirus infection	17	7.90E-03	1.80E-02
	Signaling pathways regulating pluripotency of stem cells	10	8.90E-03	2.00E-02
	cGMP-PKG signaling pathway	10	2.30E-02	4.90E-02
	Coronavirus disease - COVID-19	12	2.90E-02	6.10E-02
	Human T-cell leukemia virus 1 infection	11	4.90E-02	9.60E-02
	Spinocerebellar ataxia	7	1.40E-01	2.60E-01
	NOD-like receptor signaling pathway	8	1.70E-01	3.00E-01
	Type II diabetes mellitus	3	3.20E-01	5.00E-01
	Herpes simplex virus 1 infection	12	7.30E-01	9.30E-01
**Annotation Cluster 5**	**Enrichment Score: 2.93**	**Count**	**p-value**	**Benjamini**
	Carbon metabolism	11	5.40E-04	1.70E-03
	Glycolysis / Gluconeogenesis	8	1.30E-03	3.60E-03
	Biosynthesis of amino acids	8	2.40E-03	6.30E-03
**Annotation Cluster 6**	**Enrichment Score: 2.86**	**Count**	**p-value**	**Benjamini**
	HIF-1 signaling pathway	20	1.50E-11	4.30E-10
	ErbB signaling pathway	17	1.90E-10	3.90E-09
	Glioma	15	3.10E-09	4.10E-08
	Neurotrophin signaling pathway	17	3.10E-08	3.30E-07
	GnRH signaling pathway	13	2.70E-06	1.60E-05
	Thyroid hormone signaling pathway	14	8.30E-06	4.40E-05
	Cholinergic synapse	13	2.10E-05	1.00E-04
	Inflammatory mediator regulation of TRP channels	12	2.80E-05	1.30E-04

	Long-term potentiation	10	3.60E-05	1.50E-04
	Oocyte meiosis	13	9.10E-05	3.50E-04
	Wnt signaling pathway	14	2.90E-04	9.80E-04
	Gap junction	10	3.10E-04	1.00E-03
	Adrenergic signaling in cardiomyocytes	13	3.30E-04	1.10E-03
	Endometrial cancer	8	5.30E-04	1.70E-03
	cAMP signaling pathway	15	1.10E-03	3.30E-03
	Glucagon signaling pathway	10	1.30E-03	3.60E-03
	Oxytocin signaling pathway	12	1.50E-03	4.00E-03
	Dopaminergic synapse	11	1.60E-03	4.20E-03
	Aldosterone-regulated sodium reabsorption	6	2.00E-03	5.30E-03
	GnRH secretion	7	4.80E-03	1.20E-02
	Amphetamine addiction	7	7.00E-03	1.60E-02
	Circadian entrainment	8	1.00E-02	2.30E-02
	Melanogenesis	8	1.20E-02	2.80E-02
	Aldosterone synthesis and secretion	7	3.40E-02	7.10E-02
	Gastric acid secretion	6	4.00E-02	8.00E-02
	Long-term depression	5	6.10E-02	1.20E-01
	Insulin secretion	6	6.20E-02	1.20E-01
	Cushing syndrome	8	9.00E-02	1.70E-01
	Amoebiasis	6	1.10E-01	2.00E-01
	Endocrine and other factor-regulated calcium reabsorption	4	1.40E-01	2.60E-01
	Glutamatergic synapse	6	1.50E-01	2.70E-01
	Serotonergic synapse	6	1.60E-01	2.80E-01
	Morphine addiction	5	1.90E-01	3.20E-01
	Vascular smooth muscle contraction	6	2.40E-01	3.90E-01
	Parathyroid hormone synthesis, secretion and action	5	2.70E-01	4.30E-01
	Retrograde endocannabinoid signaling	6	3.00E-01	4.80E-01
	GABAergic synapse	4	3.80E-01	5.80E-01
	Thyroid hormone synthesis	3	5.60E-01	7.90E-01
	Salivary secretion	3	6.70E-01	9.10E-01
	Olfactory transduction	5	1.00E+00	1.00E+00
**Annotation Cluster 7**	**Enrichment Score: 1.98**	**Count**	**p-value**	**Benjamini**
	Pathways of neurodegeneration - multiple diseases	26	2.80E-04	9.40E-04
	Prion disease	15	7.70E-03	1.80E-02
	Amyotrophic lateral sclerosis	10	5.50E-01	7.90E-01
**Annotation Cluster 8**	**Enrichment Score: 1.66**	**Count**	**p-value**	**Benjamini**
	Insulin resistance	10	1.40E-03	3.80E-03
	FoxO signaling pathway	11	1.50E-03	4.00E-03
	TNF signaling pathway	10	1.80E-03	4.70E-03

	Autophagy - animal	11	2.50E-03	6.50E-03
	GnRH secretion	7	4.80E-03	1.20E-02
	mTOR signaling pathway	11	5.30E-03	1.20E-02
	Longevity regulating pathway - multiple species	6	1.80E-02	4.00E-02
	Adipocytokine signaling pathway	6	2.80E-02	5.90E-02
	Non-alcoholic fatty liver disease	9	3.80E-02	7.80E-02
	Carbohydrate digestion and absorption	4	1.10E-01	2.00E-01
	Regulation of lipolysis in adipocytes	4	1.70E-01	3.00E-01
	Longevity regulating pathway	5	1.80E-01	3.10E-01
	AMPK signaling pathway	6	1.80E-01	3.10E-01
	Small cell lung cancer	5	1.90E-01	3.30E-01
	Alcoholic liver disease	6	2.80E-01	4.40E-01
**Annotation Cluster 9**	**Enrichment Score: 0.58**	**Count**	**p-value**	**Benjamini**
	Purine metabolism	7	9.70E-02	1.90E-01
	Drug metabolism - other enzymes	4	3.20E-01	5.00E-01
	Nucleotide metabolism	4	3.50E-01	5.40E-01
	Pyrimidine metabolism	3	4.20E-01	6.30E-01
