# Supplementary material for: Proteomic Analysis of Prehypertensive and Hypertensive Patients: Exploring the Role of the Actin Cytoskeleton
Source: Int J Mol Sci. 2024 Apr 30;25(9):4896. doi: 10.3390/ijms25094896 (PMC11084483; doi:10.3390/ijms25094896)
Supplement: Supplementary file 1 [file ijms-25-04896-s001.zip › Supplementary Table S7.pdf]

Supplementary Table S7. Demographics and clinical characteristics of the participants in the validation group. Data are represented as mean ± standard deviation. One-way ANOVA was used to calculate the p-value.

	Controls	Prehypertension	Hypertension	*p*-value
**Number of participants**	25	24	17	
**Gender (M/F)**	8/17	10/14	12/5	0.043
**Age**	37.04 ± 6.6	36.13 ± 7.2	44.76 ± 6.7	<0.001
**BMI**	26.6 ± 2.4	26.3± 2.7	34.9 ± 3.6	< 0.001
**Hemoglobin g/dl**	13.03 ± 1.2	13.6 ± 1.7	14.07 ± 2	1.29
**Red Blood Cell x10^6/µl**	4.6 ± 0.6	4.9 ± 0.5	5.3 ± 0.6	0.003
**White Blood Cellx10^3/µl**	6.5 ± 1.9	6.6 ± 1.4	7 ± 1.2	0.57
**Cholesterol Total mmol/l**	5.2 ± 1	5.2 ± 0.9	5.3 ± 0.9	0.96
**LDL-Cholesterol mmol/L**	3.4 ± 0.9	3.2 ± 0.7	3.4 ± 0.8	0.62
**HBA 1C %**	5.6 ± 0.4	5.4 ± 0.2	5.9 ± 0.6	0.008
**Systolic blood pressure**	105.8 ± 7.3	128.2 ± 5.7	151.6 ± 16.2	< 0.001
**Diastolic blood pressure**	71.6 ± 6.1	86.4 ± 5.1	96.5 ± 12.2	< 0.001
**Albumin g/L**	46 ± 2.2	46.3 ± 3	44.9 ± 2.3	0.215
**ALT U/L**	20.3 ± 13.7	20.5 ± 14.3	21.3 ± 10.8	0.22
**AST U/L**	21.2 ± 10.6	20.6 ± 9.2	23 ± 5.6	0.93
**C-Reactive Protein mg/L**	4.9 ± 0.9	5 ± 0	5 ± 0	0.96

LDL: Low Density Lipoprotein. BMI: Body Mass Index. HBA1C: Hemoglobin 1C. ALT: Alanine Amino Transferase. AST: Aspartate Amino Transferase.
